# Grid Cells, Place Cells, and Geodesic Generalization for Spatial Reinforcement Learning

**DOI:** 10.1371/journal.pcbi.1002235

**Published:** 2011-10-27

**Authors:** Nicholas J. Gustafson, Nathaniel D. Daw

**Affiliations:** 1 Center for Neural Science, New York University, New York, New York, United States of America; 2 Department of Psychology, New York University, New York, New York, United States of America; Northwestern University, United States of America

## Abstract

Reinforcement learning (RL) provides an influential characterization of the brain's mechanisms for learning to make advantageous choices. An important problem, though, is how complex tasks can be represented in a way that enables efficient learning. We consider this problem through the lens of spatial navigation, examining how two of the brain's location representations—hippocampal place cells and entorhinal grid cells—are adapted to serve as basis functions for approximating value over space for RL. Although much previous work has focused on these systems' roles in combining upstream sensory cues to track location, revisiting these representations with a focus on how they support this downstream decision function offers complementary insights into their characteristics. Rather than localization, the key problem in learning is generalization between past and present situations, which may not match perfectly. Accordingly, although neural populations collectively offer a precise representation of position, our simulations of navigational tasks verify the suggestion that RL gains efficiency from the more diffuse tuning of individual neurons, which allows learning about rewards to generalize over longer distances given fewer training experiences. However, work on generalization in RL suggests the underlying representation should respect the environment's layout. In particular, although it is often assumed that neurons track location in Euclidean coordinates (that a place cell's activity declines “as the crow flies” away from its peak), the relevant metric for value is geodesic: the distance along a path, around any obstacles. We formalize this intuition and present simulations showing how Euclidean, but not geodesic, representations can interfere with RL by generalizing inappropriately across barriers. Our proposal that place and grid responses should be modulated by geodesic distances suggests novel predictions about how obstacles should affect spatial firing fields, which provides a new viewpoint on data concerning both spatial codes.

## Author Summary

The central problem of learning is *generalization*: how to apply what was discovered in past experiences to future situations, which will inevitably be the same in some respects and different in others. Effective learning requires generalizing *appropriately*: to situations which are similar in relevant respects, though of course the trick is determining what is relevant. In this article, we quantify and investigate relevant generalization in the context of a particular learning problem often studied in the laboratory: learning to navigate in a spatial maze. In particular, we consider whether the brain's well-characterized systems for representing an organism's location in space generalize appropriately for this task. Our simulations of learning verify that to generalize effectively, these representations should treat nearby locations similarly (that is, neurons should fire similarly when an animal occupies nearby locations)—but, more subtly, that to enable successful learning, “nearby” must be defined in terms of paths around obstacles, rather than in absolute space “as the crow flies.” These considerations suggest new principles for understanding these spatial representations and why they appear warped and distorted in environments, such as mazes, with barriers and obstacles.

## Introduction

The rodent brain contains at least two representations of spatial location. Hippocampal place cells fire when a rat passes through a confined, roughly concentric, region of space [1], whereas the grid cells of dorsomedial enthorhinal cortex (dMEC) discharge at the vertices of regular triangular lattices [2]. Behaviorally, such codes likely support decisions about spatial navigation [3]–[7], and more particularly reinforcement learning (RL [8]) or learning by trial and error where to navigate.

Here we investigate the appropriateness of the brain's spatial codes for learning value functions, guided by the influential use of RL models across many varieties of decision problems in computational neuroscience [9]–[11]. Although much work in these systems tends to focus on the “upstream” mechanisms by which place or grid fields are constructed from different sorts of inputs, we focus instead on learning downstream from these representations (e.g., where place cells synapse on striatal neurons), to ask what does this function suggest about or require from the spatial representations. This provides a complementary perspective on aspects of the neural responses, which, we argue, are well adapted to support reinforcement learning.

Importantly, this exercise views the brain's spatial codes less as a representation for location per se, and instead as basis sets for approximating other functions across space. In particular, most RL models work by learning to represent a *value function* over state space – a mapping of location to value. The value function measures the proximity of locations to rewards, and in this way can guide navigation towards reinforcement. Although a frequency-domain Fourier basis (often analogized to the grid representation [12], [13]) and a space-domain impulse basis (an idealized place map) are both complete representations for arbitrary functions over space, efficient RL—in the sense of rapid generalization from few experiences—depends on the features of the basis being well matched to the function being learned [14]–[17]. For instance, just as efficient visual representations are motivated by the fact that the Fourier decompositions of natural images have most of their power at low frequencies, so also value functions tend to change smoothly across space: if a given location is near reward, then so are nearby positions.

Thus, it is intuitive (and our simulations, below, verify) that low-frequency basis functions can speed up spatial RL by allowing experience about rewards to generalize over larger distances. However, we argue that considering generalization in the RL setting suggests a crucial and underappreciated refinement of this idea: in general, value functions are *not* maximally smooth over space “as the crow flies” (i.e. Euclidean distance). Instead, value functions exhibit discontinuities at obstacles, such as walls, which help to guide navigation around them. Building on a variety of work applying graph-theoretic distance metrics to different problems in machine learning [14], [15], [17], [18], much work in reinforcement learning [14]–[17] suggests that the demand of efficient generalization for navigation implies that basis functions—here, place or grid fields—should modulate their strength according to geodesic distance (i.e. the shortest navigable path between two points, around obstacles) rather than Euclidean.

We formalize this idea in a model of grid and place cell responses. The model and its simulations suggest novel predictions about how grid cell and place cell firing fields should behave in the presence of obstacles and other navigational constraints: in effect, these should locally warp the geometry of the representation. These predictions offer a new perspective on existing results, such as the unidirectionality of place fields on the linear track [19]–[23] and the behavior of grid cells in mazes [24].

### Background and previous work

#### Place cells and grid cells

Pyramidal neurons in the rat hippocampus have long been known to have firing fields in localized areas of space [1], [25], [26]. While much research has studied hippocampal neurons with small place fields [3], [27]–[29] (e.g., roughly the size of a rat) a range of place field scales have been reported [30], [31]. Recently, electrophysiological recordings from a long linear track suggest that place cells in area CA3 are multiscale, with size ranging up to approximately 10 meters at the ventral pole of the hippocampus [30]. In addition, it has been previously shown that changing environmental geometry can alter the electrophysiological characteristics of place cells [32]. The scale of the place fields was topographically organized in a manner parallel to changes in scale of the afferent grid cell input [30].

Grid cell neurons in dorsomedial entorhinal cortex, a principal input to the hippocampus, have firing fields whose hallmark is a regular triangular lattice [2]. Furthermore, grid cells show a variety of orientations, phases, and scales, with the relative size varying topographically from small to large along the dorsomedial to ventrolateral axis of the entorhinal cortex [2], [33]. Interestingly, the regularity of the firing field lattice can compress or expand under changes in the recording enclosure's aspect ratio [34], which shows their firing fields are malleable with respect to the environment's configuration, similar to findings with place cells.

#### Models of entorhinal grid cell

The discovery of grid cells spurred a great deal of computational modeling, mostly targeted at understanding their *inputs* and *outputs*. Specifically, much work considers how the characteristic triangular lattice grid cell firing fields arise [12], [35]–[42] and how they might, in turn, serve as an input representation for producing the spatially localized place fields of hippocampal neurons [12], [13], [43]–[46]. Apart from these *representational* questions, the primary *functional* question examined in grid cell modeling concerns how the cells might participate in a circuit for path integration [35], [37]–[40], [47]. The present work considers a distinct, albeit nonexclusive, role for both grid and place cells as potential basis sets for representing value functions in spatial reinforcement learning. In the case of the grid cells, this draws on the work of several authors [e.g. 12], [13], [48] who note an analogy between the multiscale, oscillating grid cell basis and a sinusoidal Fourier-like basis.

#### Models of RL in the brain

A great deal of modeling work in neuroscience and psychology concerns the brain's mechanisms for RL, founded on the observation that dopaminergic neurons in the primate midbrain appear to carry a reward prediction error signal as used in temporal-difference (TD) RL algorithms [9]–[11]. A typical architecture [e.g., 49] presumes that cortical neurons provide sensory or *state* information; striatal neurons learn to map this representation to a *value function* via dopaminergically gated plasticity at the corticostriatal synapse. In such models, the cortical “state” representation provides a linear *basis* for representing the value function: values in striatum are estimated as weighted sums of cortical inputs. In the context of spatial tasks [3], [50], [51], it is typically assumed that the relevant striatal subregion is the nucleus accumbens, which is involved in locomotion [see 4] and that the state input arises from the hippocampal place code.

Here, we revisit this architecture, focusing on the role of both the hippocampal and entorhinal spatial codes as bases for building the value function, in order to connect neural observations to work in RL on advantageous representations for value function approximation [14]–[17]. The main questions we investigate concern the generalization properties of spatial basis functions, and specifically how RL performance is affected by the distance metric (Euclidean or geodesic) over space that they embody. To illustrate the generality of these geometric ideas, we simulate our Euclidean and geodesic models under the standard assumption that place cells serve as the spatial representation for downstream value function learning, and also show that the same geometric conclusions hold even when taking the grid cell representation, which have quite differently behaved firing fields, as a direct basis for value learning. The latter hypothesis is clearly more speculative, and would depend on the existence of direct projections from the grid cells to the site of value learning, likely nucleus accumbens, as well as those via hippocampus. Grid cells are most commonly reported in the superficial layers (II–III) of dMEC, which project directly to hippocampus [52] though they have also been reported in deep layers [53], [54], where intracortical and subcortical projections originate. Moreover, there is anatomical evidence of projections from entorhinal cortex to nucleus accumbens [53], [55]–[57], with some connections possibly originating from areas near those where grid cells are found [57]. Finally, lesions in both areas demonstrate an involvement of entorhinal cortex, not mediated via hippocampus, on instrumental (albeit, in this case, not spatial) learning [58]. Note that our model's geometric predictions about how the grid cell representation should behave do not depend on the idea that it serves as a direct substrate for value learning: since the grid cell representation is thought to serve as a precursor of the place cell representation (though see [59], [60]), it would be likely to share the same geometry (geodesic or Euclidean) with that representation in any case.

## Results

### Euclidean grid cell and place cell like basis functions

**Figure 1:**
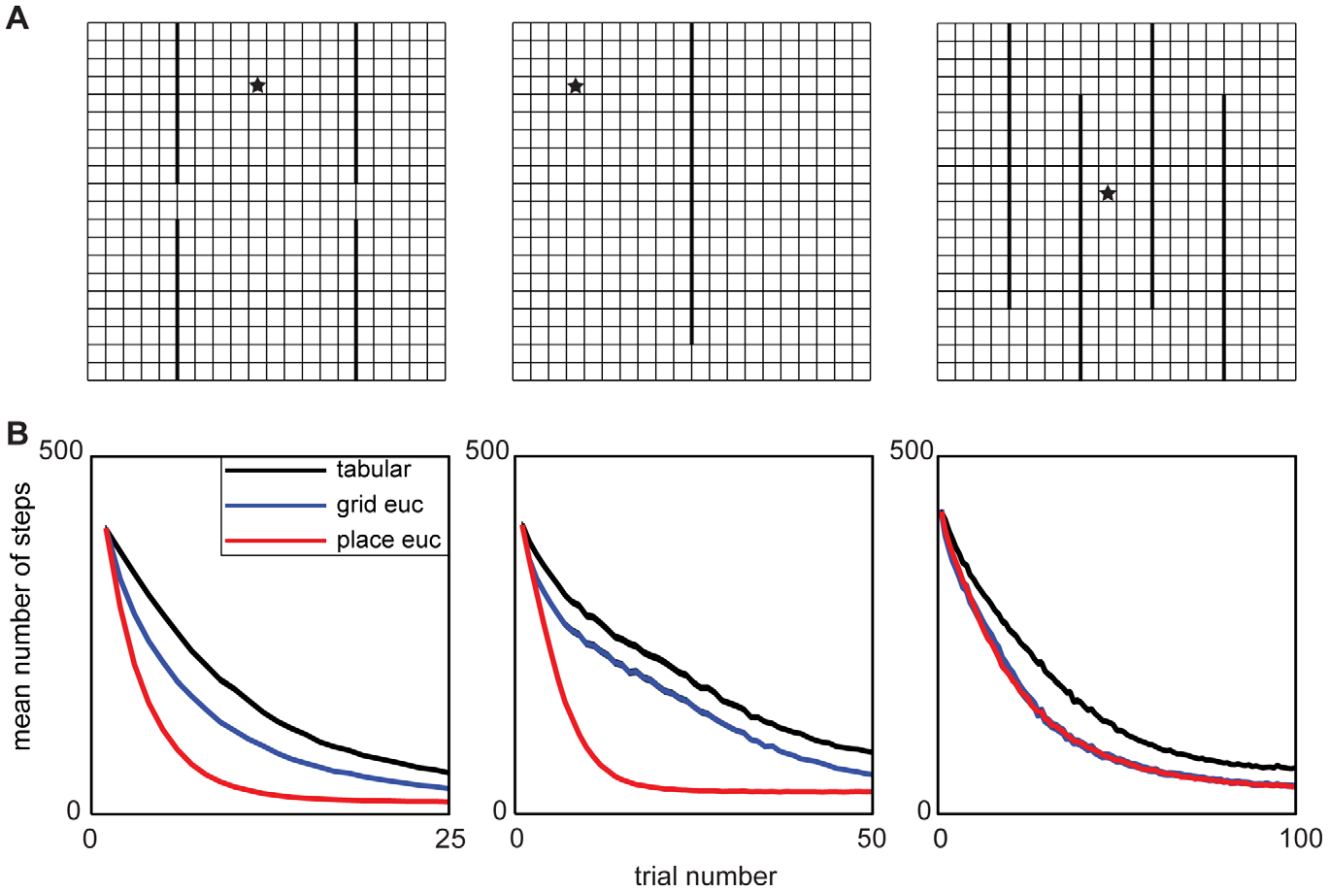
Euclidean spatial generalization benefits learning in simple navigation tasks. (A) Each column displays the gridworld configuration whereby individual squares are discrete states, thick black lines are walls, and the star indicates the goal state with reward of 1. (B) Each column shows performance measured as the mean number of steps to goal over 10,000 runs for the environment in the corresponding column in A. The width of each line occupies at least the 95% confidence intervals on the means (range 3.9–4.4 steps). Within a given gridworld the different colored lines represent different basis sets with black for tabular, blue for grid cells, and red for place cells.

First, we used TD(λ) learning in three simple environments (Figure 1A) to test the ability of multiscale grid cell- and place cell-like basis sets to learn value functions in spatial RL (see Materials and Methods). In order to verify the importance of generalization over long spatial scales, we compared learning with the modeled grid and place cell bases to a standard, tabular RL basis learning the same task. This is like a place cell basis using only a single, fixed scale of representation that is small with respect to the task-relevant distances. The simulated agent had to learn to navigate from a randomly chosen starting point to a goal state that contained a reward. To quantify performance, the number of steps needed to reach the reward was plotted as a function of the training trial. Although our key qualitative points are robust to changes in the free parameters (simulations not shown), to ensure a fair comparison we optimized the learning rate (a crucial free parameter) separately for each condition (i.e. basis function and gridworld) to obtain its best performance. We additionally used the TD(λ) generalization of TD with a high value (0.9) of the eligibility trace parameter λ, since this provides another mechanism for learning to generalize along trajectories and might, in principle, help to compensate for the shortcomings of the tabular or Euclidean bases.

**Figure 2:**
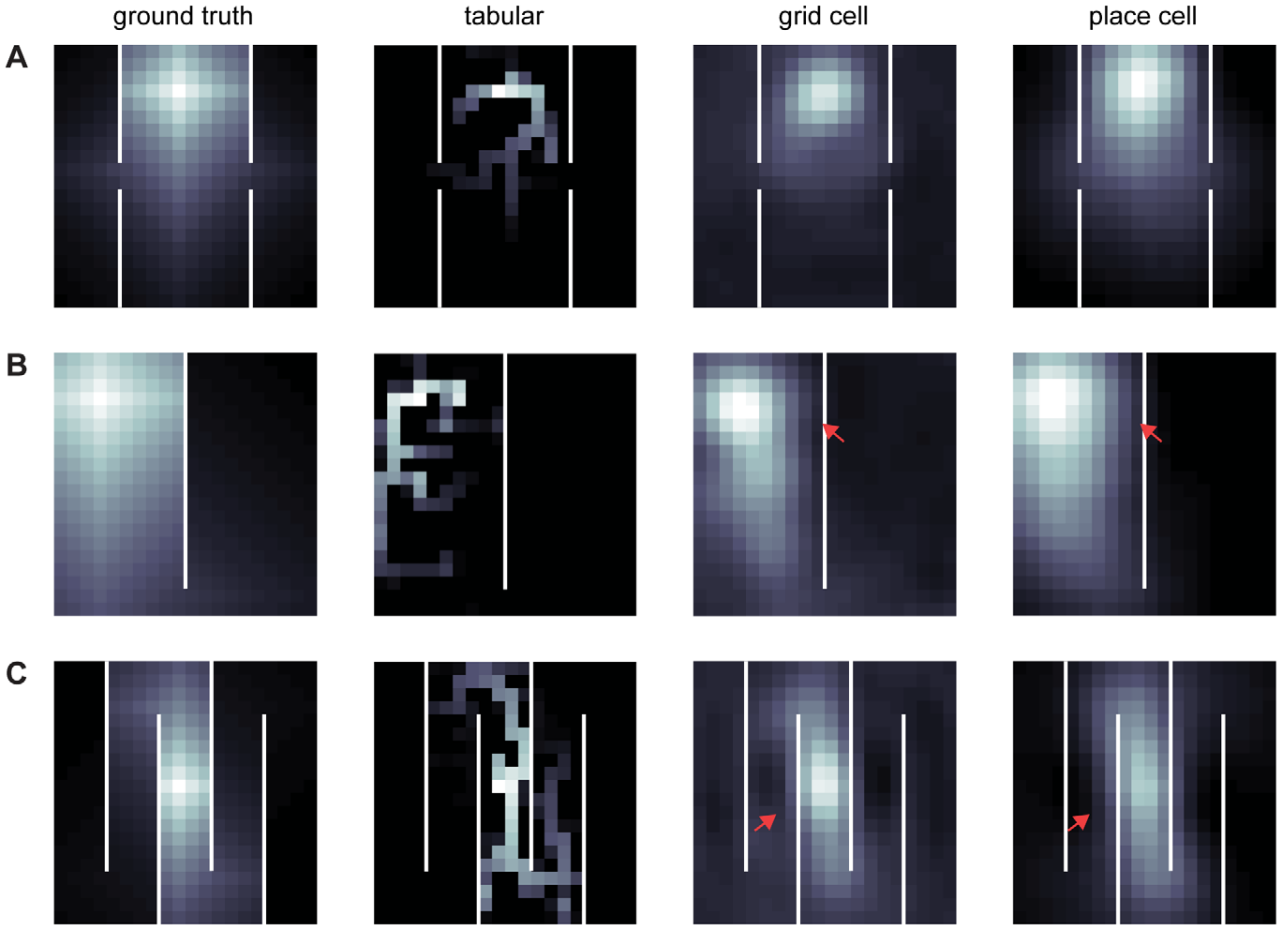
Qualitative comparison of learned value functions using tabular, Euclidean grid cell, and Euclidean place cell bases. In each figure A–C, the column titles indicate the representation used to learn the value functions for a given gridworld configuration, and each row corresponds to an environment. White lines are walls, discrete squares indicate states, and the gray scale from dark to light indicates low to high value, respectively. To ease comparison between spatial representations within a given gridworld, the image brightness was normalized with respect to the optimal value function. (A) Snapshot of value representation after 15 learning trials. (B) Snapshot of value representation after 25 learning trials. (C) Snapshot of value representation after 50 learning trials. Notice that for both grid cells and place cells, the value representation bleeds across walls, indicated by red arrows where the estimated value is too low (relative to ground truth) on the side of a wall nearer a reward or too high on the far side.

As Figure 1B shows, the grid and place cell basis sets drastically quicken learning the value function compared to the tabular code, demonstrating the benefits of spatial generalization. Figure 2 illustrates the approximated value functions at different stages of learning and qualitatively shows the importance of generalization. In particular, the tabular basis does not take advantage of the spatial structure to generalize quickly and must learn each state's value separately from its neighbors by a slow process of TD chaining.

Figure 2 also hints at a subtler problem of overgeneralization in Euclidean space. In particular, these grid and place cell basis functions tend to smear the value function across barriers, where it should change sharply (arrows in Figures 2B and 2C, where the effects are most apparent). Because of this, value is underrepresented at states inside the walls (i.e. locations closer to the reward, as in 2B) and overrepresented on the other side of the barrier (most visible in 2C). This distortion remains at asymptote and is likely not an artifact of insufficient experience.

**Figure 3:**
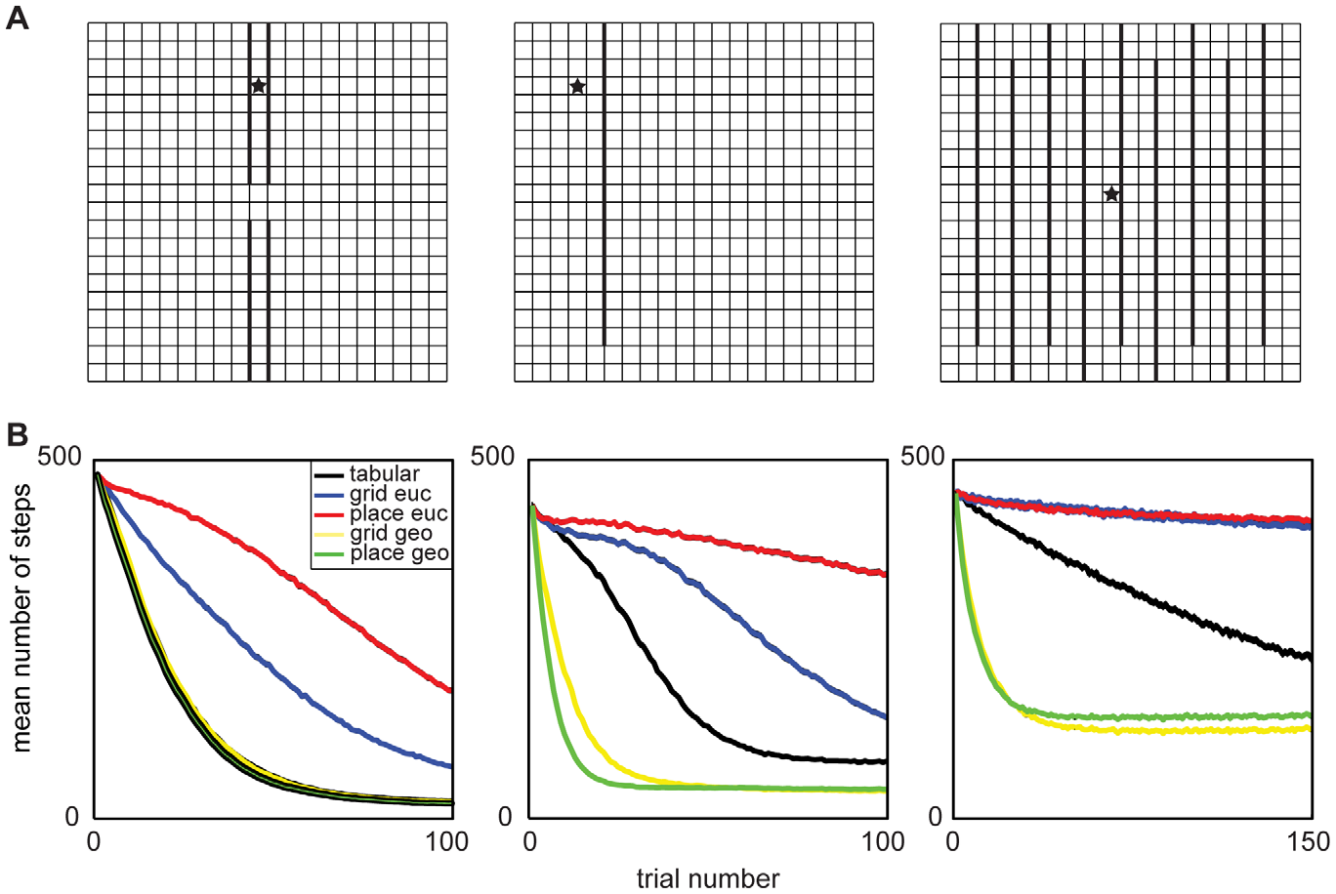
Geodesic representation required for learning when value function has sharp discontinuities in Euclidean space. (A) Each column displays the gridworld configuration whereby individual squares are discrete states, thick black lines are walls, and the star indicates the goal state with reward of 1. (B) Each column shows performance measured as the mean number of steps to goal, over 10,000 runs for the environment in the corresponding column in A. The width of each line occupies at least the 95% confidence interval on the means (range 3.2–4.5 steps). Notice that the collapse of learning, present in the Euclidean grid cells (labeled *euc*) and place cells (blue and red), is recovered by their geodesic counterparts (labeled *geo*, yellow and green, respectively).

While this flaw does not notably degrade performance in these simple tasks, it can be detrimental when fine navigational precision is required. To demonstrate this, we tested the models in three environments that required the agent to navigate narrow halls or openings, and thus learn precise state value representations (Figure 3A). Here, the grid cell and place cell basis functions performed poorly, and were outperformed by the tabular basis (Figure 3B). Together, then, these simulations demonstrate that generalization due to spatial representations like those seen in the brain can help make reinforcement learning more efficient, but also that such generalization has drastic (and, presumably, behaviorally unrealistic) side effects, abolishing learning in tasks where paths are narrow.

### Geodesic grid cell and place cell like basis functions

In general, as can be seen directly in the recursive definition of the value function, (Equation 1 in Materials and Methods), the extent to which values are related between two states depends on how closely they are connected by the state-state transition probability function. Accordingly, work on value function approximation for reinforcement learning has proposed [14]–[17] that basis functions should be constructed to respect distance along the state transition graph. For instance, in temporal prediction tasks, value functions are smooth in time [61]. In a spatial task, the transition dynamics imply that states have similar values when they are near each other, but near as measured in geodesic (along-path) distance, rather than “as the crow flies” (Euclidean distance). Formally, geodesic distance measures the number of steps along the transition graph needed to get from one state to another. A basis over geodesic distances would treat states separated by a boundary as comparatively far apart, enabling their values to be discontinuous, whereas the Euclidean basis used above (and ubiquitously to characterize the spatial extent of place and grid fields) would inappropriately treat them as adjacent.

These considerations suggest that for efficacious representation of value functions over state space, the brain should adopt basis functions that are smooth along geodesic rather than Euclidean distances. In the open field there should be no difference between geodesic and Euclidean representations, since these metrics coincide there. However, if an environment has barriers, then Euclidean and geodesic firing fields will differ. The effect of such a difference should be to introduce geometric distortion into geodesic firing fields nearby obstacles, where geodesic and Euclidean metrics differ. Such a distortion can be characterized (and indeed implemented) by mapping the original Euclidean vector coordinates through an additional transform that accounts for geodesic distance. However, in the present work our goal is to investigate the brain's spatial representations through the lens of their downstream computations; thus, in contrast to much work on the hippocampal system [12], [35], [36], [40], [43], [44], [47], [48], [62], [63] we do not focus on the “upstream” computations by which the grid or place representations (or their hypothesized distortions) are themselves computed from inputs. That is, we take geodesic or Euclidean representations as a given and focus our analysis on hypothesized learning that relies on entorhinal and hippocampal outputs.

**Figure 4:**
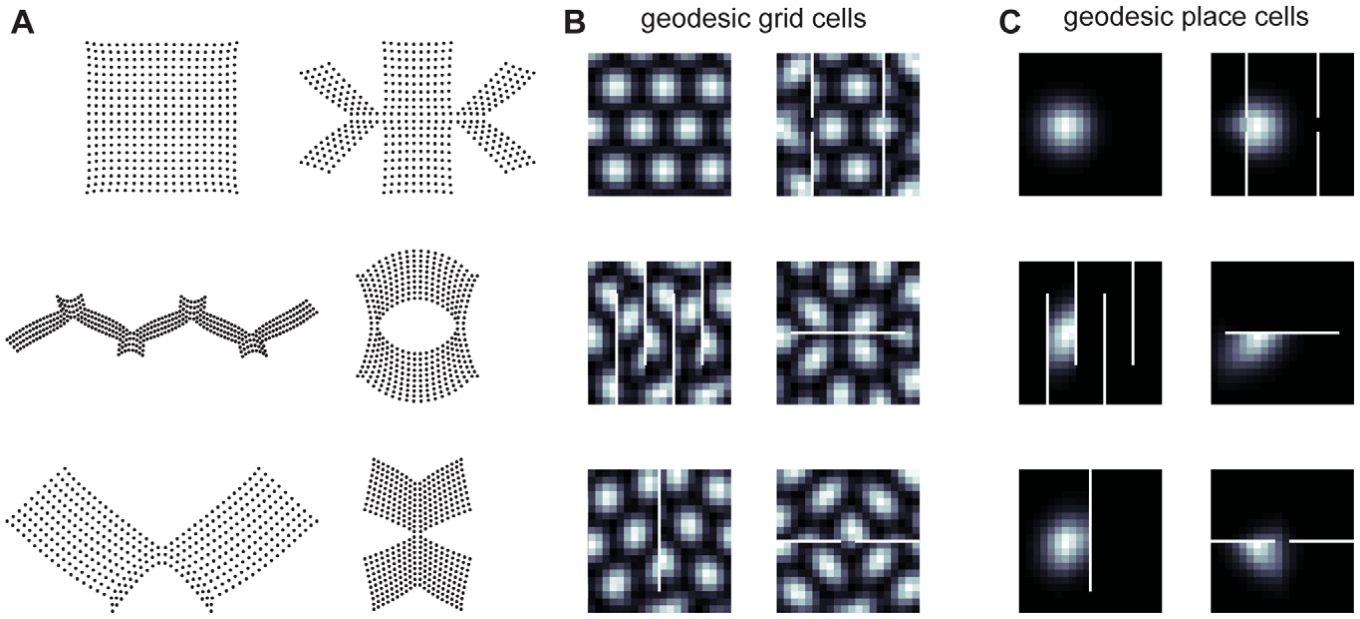
Example geodesic transformations of grid cells and place cells. (A) Geodesic coordinates for different environments. (B) Single grid-cell using respective geodesic coordinates. Each grid cell generated using the same spacing, orientation, and relative spatial phase. (C) Single place-cell using respective geodesic coordinates. Each place cell generated using the same mean and variance.

In particular, we modeled how basis functions would appear in environments with barriers, if they followed a geodesic metric, by evaluating Euclidean grid or place fields (characterized by spatial grids or Gaussians) over a new set of x–y coordinates, chosen such that their pairwise Euclidean distances approximated the states' geodesic distances (see Materials and Methods). When viewed in the original Euclidean space, the effect of barriers is to produce geometric distortions, such as variations in grid orientation and firing field shapes (Figure 4). As one might expect, the basis functions tend not to cross walls and instead skirt along connected paths.

**Figure 5:**
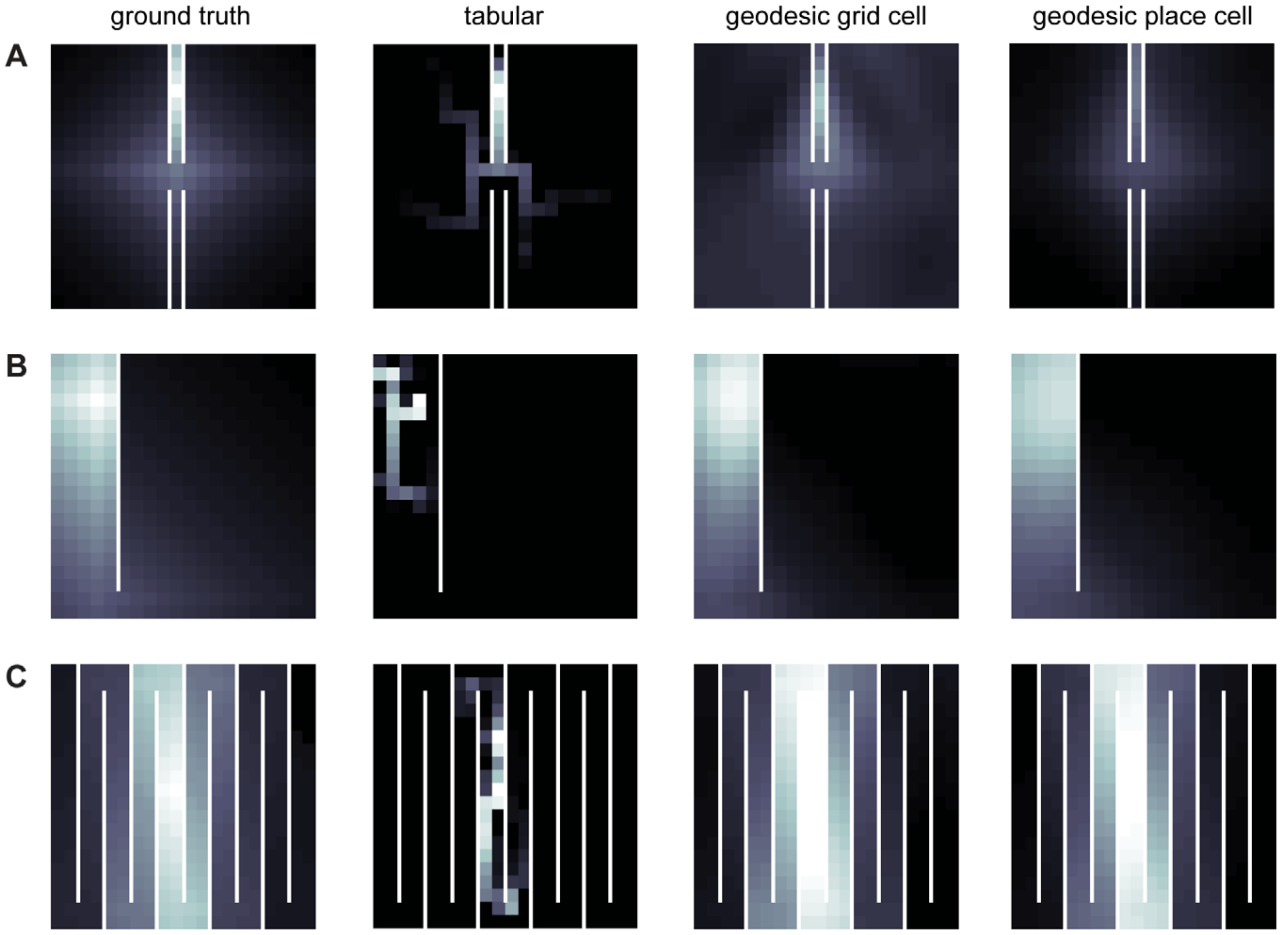
Qualitative comparison of learned value functions using tabular, geodesic grid cell, and geodesic place cell bases. In each figure A–C, the column titles indicate the representation used to learn the value functions for a given gridworld configuration (denoted by row). White lines are walls, discrete squares indicate states, and the gray scale from dark to light indicates low to high value, respectively. To ease comparison between spatial representations within a given gridworld, the image brightness was normalized with respect to the optimal value function. (A) Snapshot of value representation after 25 learning trials. (B) Snapshot of value representation after 25 learning trials. (C) Snapshot of value representation after 50 learning trials. In contrast to Euclidean bases, the geodesic representation does *not* smear value across walls but instead tracks around them.

We tested the geodesic bases in the environments that stressed importance of along-path generalization (Figure 3A). As can be seen, the geodesic bases alleviated the poor learning caused by the indiscriminate generalization of their Euclidean counterparts (Figure 3B). Since the geodesic grid cells and place cells generalize using the state transition graph, they learn at least as fast as the tabular TD control (Figure 3B). Figure 5A–C depicts typical value functions at different stages of training using the geodesic basis functions (25 trials for Figure 5A–B, 50 trials for Figure 5C). Also note that both the Euclidean and geodesic bases used the same multiple granularities and tiling, with the sole difference the distance metric used. To test the role of multiple tilings in learning, we performed follow-up simulations for each of the six gridworlds using three different tiles bases. While the tile bases often learned faster than the tabular basis (which one would expect), overall the geodesic bases tended to perform best (data not shown). Together, these simulations demonstrate the representation benefits conferred by geodesic generalization, in particular how generalization along paths rather than across walls solves the problem of overgeneralization interfering with learning in the presence of obstacles. That the same qualitative results hold up using both grid-cell-like and place-cell-like representations points to their generality. In simulations not shown here, we also produced similar results using an overlapping tile code at a variety of single scales [8], suggesting that the results relate to spatial generalization per se and not to the multiscale nature of the (biologically inspired) bases used here.

### Modeling previous grid cell and place cell data

The foregoing simulations suggest that to support efficient navigation, the brain's spatial representations should generalize according to a geodesic rather than a Euclidean metric. Of course, these two representations coincide in the open field, where most studies have been conducted. However, we believe our model's predictions are consistent with a number of studies where researchers recorded neurophysiological activity while rats foraged in environments containing barriers. Here we compare our model to examples from three studies [24], [64], [65].

**Figure 6:**
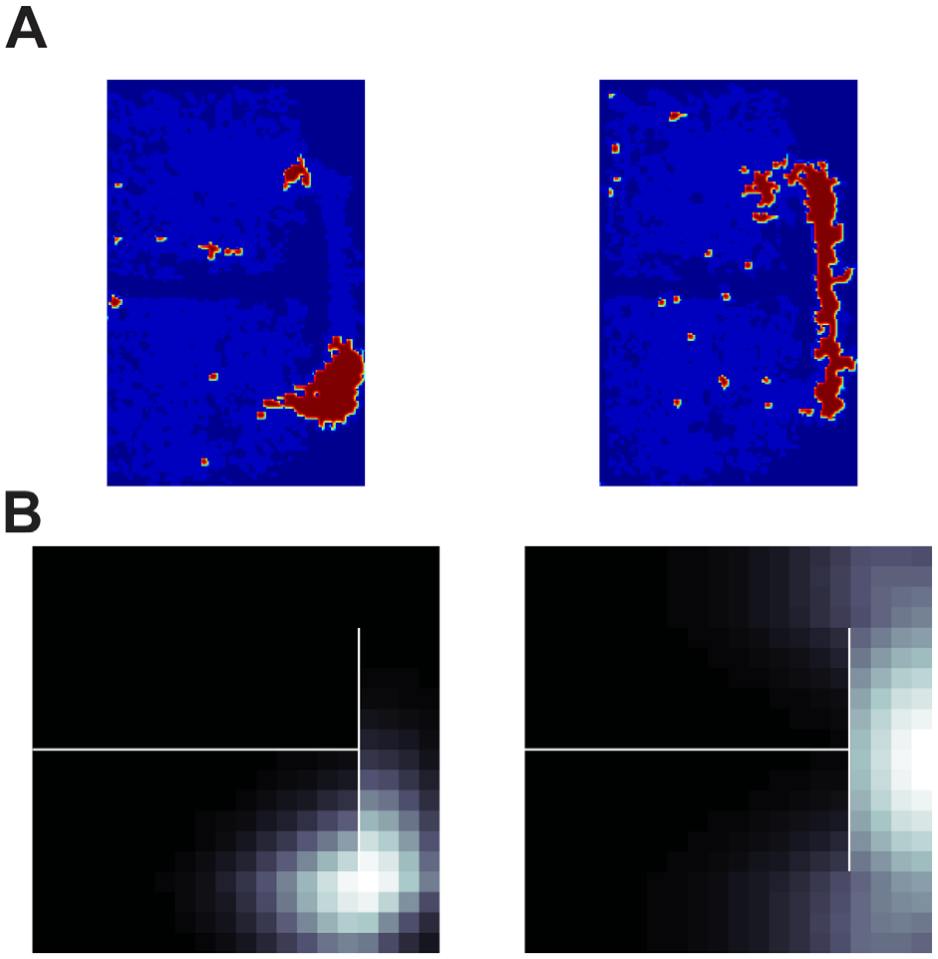
Example of geodesic place cell model qualitatively capturing recorded place cell data. (A) Data adapted and replotted from [64]. Light blue shows presence of rat, red & yellow indicate action potentials of a two hippocampal place cells, and dark blue are areas rat did not visit. (B) Simulated geodesic place cell firing fields roughly resemble data in A (*left* and *middle*). The black to white color scale represents low to high firing rates.

Skaggs & McNaughton [64] recorded place cells as rats moved between two separate enclosures that were connected by a narrow corridor (schematized in Figure 6, top; cf. Figure 4 in [64]). Although this was not the major experimental question of the study, the narrow corridor provides a good test for our model's prediction that place fields should track along paths rather than (as a Euclidean place field predicts) across barriers. In the examples reproduced here, for instance, place cell spikes are almost exclusively confined to either the connecting corridor's entrance (Figure 6A, left) or the pathway between the two rooms (Figure 6A, right). The spikes do not generalize across the walls separating parts of the environment, but instead appear to track along paths around them (Figure 6), even though a standard isotropic Gaussian place field over Euclidean coordinates would clearly not respect these barriers. The data are, however, similar to place field responses from the geodesic model in a similar environment (Figure 6, bottom).

**Figure 7:**
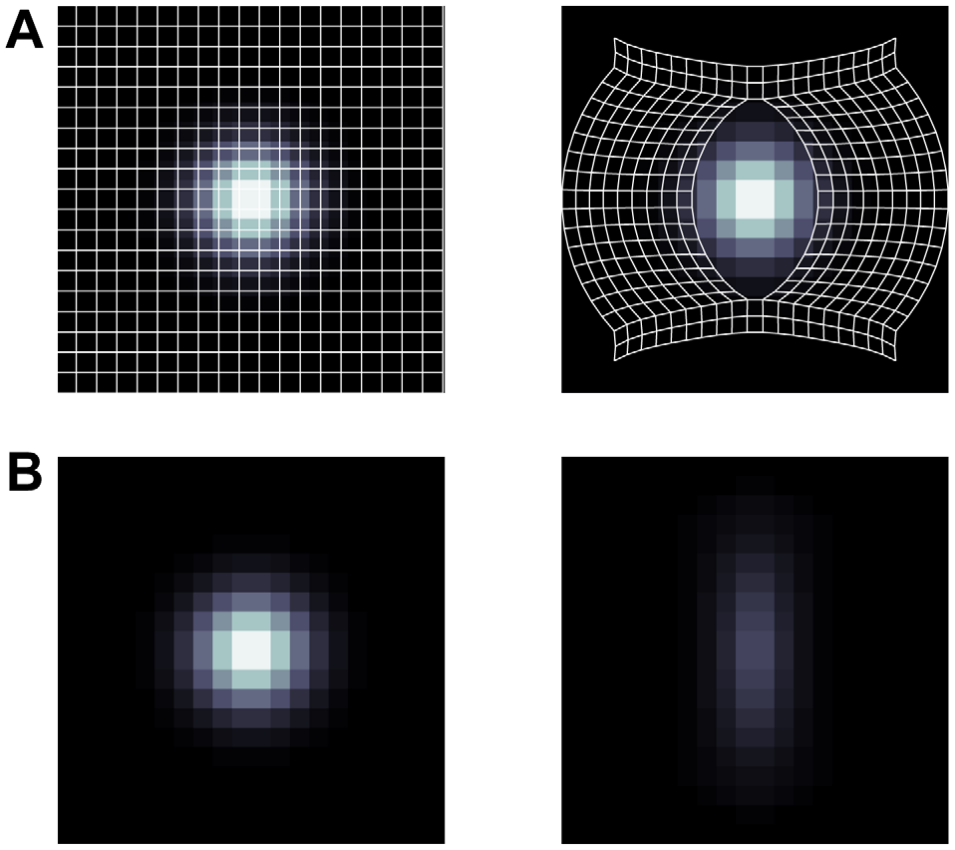
Geodesic spatial representation models can also account for the disappearance of place fields when a wall bisects the firing field. Muller & Kubie [65] observed that place fields disappeared when bisected with a wall. (A) Graphical intuition of the effect adding a wall has on the coordinates and hence the place cell firing properties. In the left panel are 20 by 20 evenly sampled points in an open square environment. Shown in the right panel are the geodesic transformed coordinates for a 20 by 20 state environment when a single vertical barrier bisects the middle section of the gridworld. Underlying each of the coordinates is a model place cell's firing field in Euclidean space (low to high firing represented by dark to light grayscale). (B) Left panel shows an open field place field, while right panel shows a geodesic place field for coordinate shown in A. Both simulated firing fields used the geodesic place cell model. Compare to Figures 8 and 9 in [65].

In another study [65], a place field was first recorded in an open box and again after adding a barrier to the enclosure (Figure 7A; cf. to Figure 8 in [65]). Recorded hippocampal place cell responses in the open field vanished immediately when the firing field was bisected by a wall [65], Figure 6A. The geodesic model of neural spatial representation provides an elegant, intuitive account for why the place field disappears, whose graphical intuition is displayed in Figures 7A–B. In an environment without walls, one can think of the recorded place cell activity being measured over evenly spaced locations in 2D enclosure (Figure 7A, left). Once a barrier is introduced that bisects the field, the nearby locations on adjacent sides of the wall are pulled apart, which changes the spacing between neighboring points compared to its Euclidean counterpart. Locations on either side of the wall are far, in geodesic terms, from each other, and from the center of a place field centered in the wall itself. As a result, a sinkhole is created that swallows the place field in the geodesic coordinate space, thus muting its activity (Figure 7A–B).

**Figure 8:**
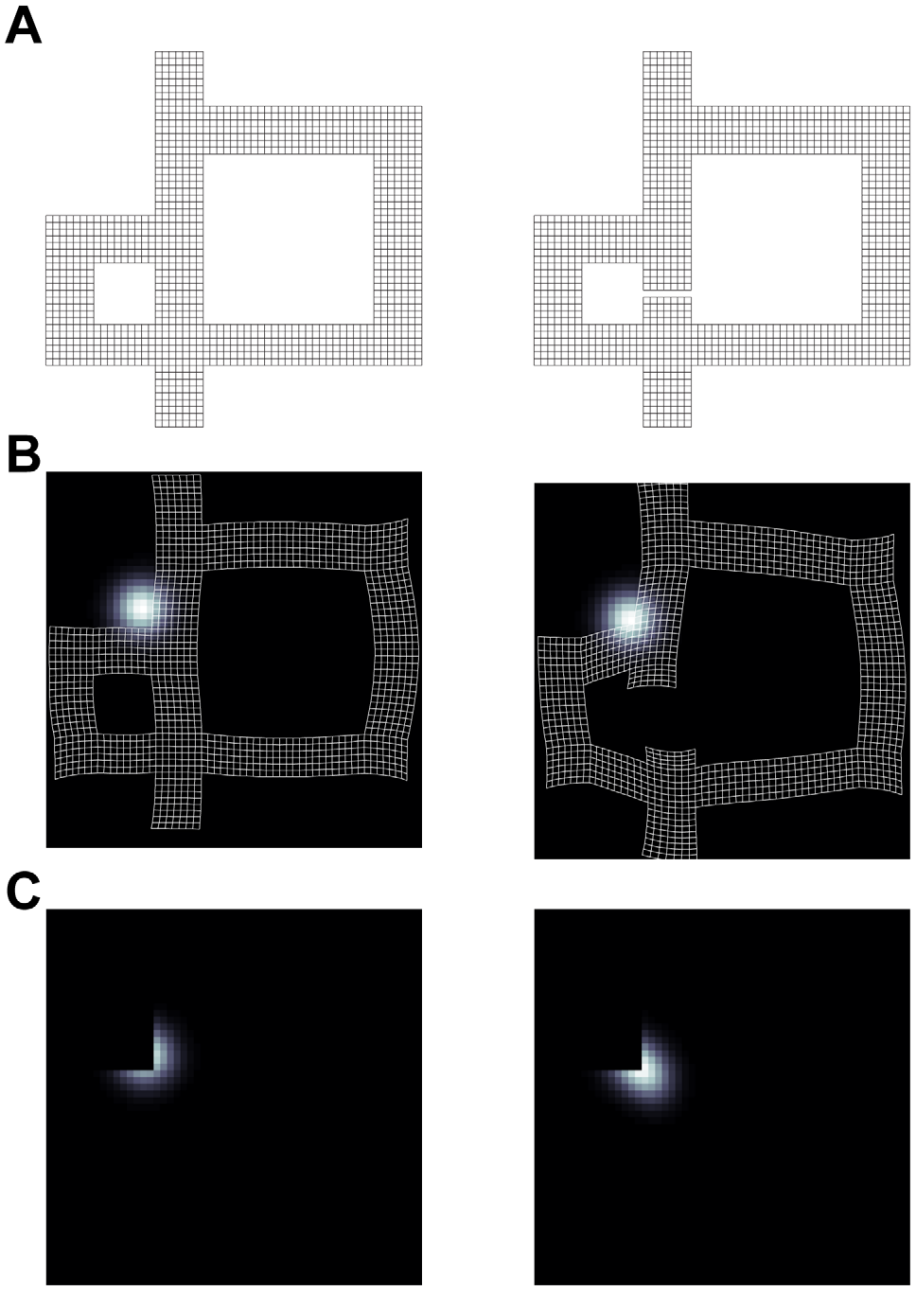
Example of geodesic place cell model qualitatively capturing recorded place cell data. (A) Two example environments used in [66]. Maze on the left was used for training & exploration and maze on the right was used for testing whether the rat learned to take the shortcut route. (B) Geodesic embedding of mazes shown in A. Underlying each of the coordinates is a place field. (C) Example place field computed using coordinates shown in B; the place field center and half-width was the same in each condition. The geometric distortion in the coordinates introduced the wall can lead to increased activity in the geodesic place cell model.

Similar results were also seen in a recent study of how place cell firing fields changed when mazes were reconfigured [66]. In particular, this work replicated the phenomenon of place fields diminishing or disappearing near newly introduced obstacles, and verified (as in our simulations) that such changes predominate near newly introduced obstacles. The study also demonstrates a rarer, complementary phenomenon whereby the introduction of obstacles caused firing to increase or even new place fields to appear, as verified in our simulations. In our model (Figure 8), increased firing is the flip side of responses diminishing for neurons coding “holes” in geodesic space; it occurs when geometric distortion “pushes” locations into areas previously off the map.

**Figure 9:**
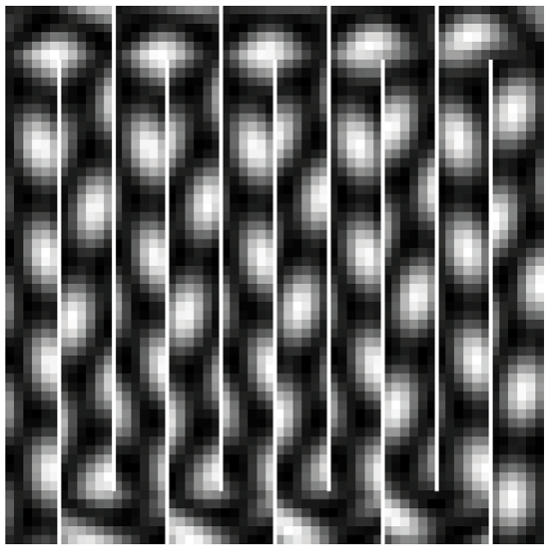
Example of geodesic grid cell model qualitatively capturing recorded grid cell data. Derdikman et al. [24] recorded while a rat explored a hairpin maze and observed fractionated grid cell firing fields that were phase locked to alternating arms of the maze. Shown is an example geodesic grid cell firing field for a similar hairpin maze that resembles that used in [24]. The black to white color scale represents low to high firing rates.

Finally, Derdikman et al. [24] recorded from grid cells as a rat ran along a hairpin maze. Figures 1 and 2 from [24] show typical grid cell firing fields in an open field and again in a hairpin maze. The standard hexagonal pattern of responding is extremely distorted; instead, responses tend to track along the hallways but not to cross walls, and firing fields are similar between alternate arms. Grid cells simulated in the geodesic space share a number of these characteristics (Figure 9), though not (as discussed below) all of them. One limitation of the model is that it does not capture the repetitive place field firing observed by Derdikman et al. [24].

## Discussion

Although researchers widely assume that reinforcement learning methods such as temporal difference learning subserve learned action selection in the brain [9]–[11], it is less clear how tasks involving many structured states can be represented in a way that enables these methods to learn efficiently, due in large part to the curse of dimensionality. In computer science, stylized spatial navigation (gridworld) problems are the classic domain for studying this issue, since the state space is large but transparently visualized and manipulated [8]. Here we consider rodents' neural representations of spatial location from this perspective, treating them as basis functions for downstream reinforcement learning in high-dimensional state spaces and asking how well adapted they are to this role. Though previous modeling work has not extensively considered the constraints on the brain's location codes implied by this function, much work has more or less implicitly exploited the idea that unlike the tabular basis often assumed in simple RL, the spatial extent of place fields can help to cope with the curse of dimensionality by allowing learning to generalize between nearby locations [3], [50], [51] even over multiple scales [30].

The present study extends this idea to consider such generalization in light of work on efficient representation in machine learning [14]–[17]. These theoretical considerations, illustrated and verified by our simple simulation results, suggest that to enable efficient representation of value (or other) functions over space, grid and place fields should operate in a distorted geometry: generalizing according to geodesic (on-path) rather than Euclidean (as-the-crow-flies) distances. Although these two distance metrics coincide in the open field, they differ in the presence of boundaries. The geodesic metric predicts that grid and place fields should not spill across walls but should instead track along paths, and should also exhibit geometric distortions, such as altered grid orientation, near boundaries. We have reviewed data from a number of experiments that seem largely in accord with these predictions. It should be noted that these predictions are all at the neural level, and could be most directly tested quantitatively by simply examining whether neural firing is modulated more reliably with distances measured by either metric: e.g., regressing distance (computed according to either definition) from a place field's center on firing rate.

By contrast, since our argument is primarily one about learning efficiency (which is difficult to quantify behaviorally, since it is affected by many factors), our model does not make categorical behavioral predictions. Our simulations (Figure 3) demonstrate that simple TD models with Gaussian place fields (like that of [3]) can entirely fail to solve simple navigation problems involving narrow apertures or hallways. However, the fact that rats do not exhibit such problems of course does not by itself demonstrate that the brain adopts the same solution for this problem as the one we propose. Also, to focus on our main questions of interest, we omit many features that other models use to explain various behavioral phenomena of navigation, among them mechanisms for allocentric route-planning (important for quick goal learning [3] and for planning shortcuts [67]) and localization driven by combinations of cues and path integration [4], [68], both issues we discuss further below.

The concept of geodesic generalization provides a formal perspective on spatial representation which is different from, but complementary to, much other work in this area. Whereas much experimental and theoretical work on the hippocampal formation concerns essentially sensory-side questions—how place or grid cells combine different sorts of inputs to produce their instantaneous representations, or to learn them over time—we attempt to isolate the downstream question of how the resulting representations serve downstream learning functions. To this end, we do not address the input-side question of how the hypothesized distorted spatial representations are themselves produced from more elementary inputs. We only assume, abstractly, that the basis functions are computed on the fly from a learned map of the barriers in the environment. In sparse environments such maps could easily be learned from observation in a single trial, and may implicate the “border cells” of entorhinal cortex [69]. All this leaves open the opportunity, in future work, for studying how the input- and output-side perspectives relate: whether the mechanisms studied by previous authors might be made to produce or approximate representations of the sort we envision. For instance, in the geodesic view, place fields tend to be unidirectional on the linear track [70], [71] because the states of passing through them facing either direction are far apart in the state transition graph of a shuttling task. In input terms this more abstract relationship between states may be reflected in these situations being visually distinct [70], [71] or anchored to a different prior reference point [72].

More generally, unlike idealized RL models [3], [51], theories of how place cells arise from sensory inputs (e.g. via competitive learning [70], [71], or self-organizing maps [73]) do not necessarily imply the isotropic Gaussian firing fields we criticize, and thus may also offer (more mechanistic) explanations for phenomena such as place fields not crossing walls. It remains to be seen to what extent such local learning rules can be massaged to produce maps that accord with the globally geodesic ideal. However, such unsupervised learning models tend to envision that representations are acquired incrementally over time, which stands in contrast to our assumption (supported by data such as place field changes occurring immediately when barriers are added [65]) that the geodesic basis is computed on the fly with respect to the current barrier locations. A different mechanism that could be useful in producing geodesic firing fields is the “arc length” cell posited by Hasselmo [63], [74], a circuit for computing along-path distance using oscillatory interference mechanisms related to those thought to be involved in grid formation. This mechanism has already been used to explain several examples of context-dependent firing of hippocampal neurons similar in spirit to the phenomena we consider here.

The behavior of the entorhinal representation also raises interesting questions about the relationship between input- and output-side considerations. To start, it is often assumed that the place code is built up by linear combinations of grid cell inputs, e.g. by a sort of inverse Fourier transform [13]. In such a model, it can be shown (and simulations, not shown, verify) that place cells will inherit the geometry of their grid cell inputs. For this reason, we suggest that grid cells are likely to use a geodesic metric even if they do not directly serve as a basis for value function learning (but only indirectly, as a basis for geodesic place cells). However, this exposes some tension between the output-side imperative of generalization for RL, which we have argued calls for geodesic distortions, and the input-side implication of the system in path integration (i.e. tracking vector coordinates in a path-independent manner) [35], [37]–[40], [47], [75], which is an inherently Euclidean operation.

In this respect, the recent results of Derdikman et al. [24] showing distorted and fractionated grid fields in a hairpin maze seem difficult to reconcile with a global Euclidean path integrator (since the hairpin barriers do not change the Euclidean coordinates), and at least qualitatively more in line with the geodesic view. One possible path toward reconciling these considerations is to consider a sort of hierarchical representation that treats the environment as a collection of rooms (in the hairpin maze, hallways) whose interrelationships are represented as by a geodesic graph, but with (disjoint) Euclidean representations maintained within each of them. This has resonance with multi-level navigation models from animal behavior (e.g. [68]), with multiple map views of hippocampus [72], and, also, mechanistically, with some of the more detailed aspects of the Derdikman [24] data that are not captured by our model. Most importantly, the Derdikman data suggest that the grid phase resets and “anchors” at left or right turns, producing similar patterns in alternating arms and suggesting a possible mechanism for separating adjacent hallways' representations. Such heuristics for grid resetting and anchoring (and also stretching) [24], [34] may be able to produce a “good enough” approximation to the geodesic metric, at least in some environments, and have been examined in much more detail in more biologically detailed modeling of the task [38]. One sign of approximations is where they break down. In this respect, it is interesting that the rather extreme case of the hairpin maze results in badly fractionated downstream place fields as well [24], a phenomenon not predicted by the exact geodesic model. Finally, unlike our full model, a resetting mechanism would not in itself seem to explain phenomena related to barriers within a room, such as those we illustrate in Figure 7. A fuller understanding of these sorts of mechanisms demands additional research, both experimental and theoretical.

Our simulations also demonstrate that the grid representation itself is a suitable basis for value function learning, even without an intermediate place cell representation. On one level, these results serve to underline the generality of our points about geometry and generalization, using a rather different basis. More speculatively, they point to the possibility that the grid representation might actually serve such a role in the brain, echoing other work on the usefulness of this Fourier-like basis for representing arbitrary functions [12], particularly (as also for standard uses of Fourier representations in engineering for compressing images and sounds) smooth ones. However, although a few studies have demonstrated anatomical connections from the entorhinal cortex to striatum [55]–[57], [76], grid-like responses are less often reported in the deep layers that give rise to these subcortical projections (though see [53], [54]).

Finally, although for simplicity and concreteness we have focused on the principles of value function generalization in the context of a particular task (spatial navigation) and algorithm (TD(λ) learning), many of the same considerations apply more generally. First, across domains, in computational neuroscience, the need for (temporally) smooth basis functions been suggested to improve generalization also in learning about events separated in time rather than space [61], though there is no obvious counterpart to the geodesic distance metric in this setting.

Second, across algorithms, TD-like learning mechanisms also likely interact with additional ones in the brain, and the core considerations we elucidate about efficient generalization due to appropriate state space representations crosscut these distinctions. For instance, value functions may also be updated using replay of previously experienced trajectories (e.g., during sleep) [28], [51]. In models, this is typically envisioned to operate by the same TD learning rule operating again over the replayed experience [51], [77], and thus should imply parallel considerations of efficiency with respect to the number of replayed experiences required for convergence depending on the generalization characteristics of the basis. More distinct from these models, since the work of Tolman [67] it has been believed that spatial navigation may in part be accomplished by map-based route-planning processes that in RL terms correspond to model-based algorithms [78]–[82] rather than model-free algorithms like TD learning. These algorithms plan routes from a learned representation of the state transition matrix and rewards, typically using variants of the value iteration algorithm to compute state or action values. The core of this process is the iterative evaluation of Bellman's equation (Equation 1 in Materials and Methods), the same equation sampled with each learning step of TD. Thus, there is reason to think that efficient value iteration (here defined as fast convergence of the value function over iterations) will analogously occur when the update is over state representations that provide better generalization over states at each step. In all, then, although we exemplify them in a highly simplified model, the principles of state representation for efficient reinforcement learning are quite general.

Another issue arises when considering the present model in light of model-based RL. One of the hallmarks of model-based planning (and the behavioral phenomena that Tolman [67] used to argue for it, albeit not subsequently reliably demonstrated in the spatial domain), is the ability to plan novel routes without relearning, e.g. to make appropriate choices immediately when favored routes are blocked or new shortcuts are opened. Interestingly, rather than by explicit replanning, some such behaviors could instead be produced more implicitly by updating the basis functions to reflect the new maze, while maintaining the weights connecting them to value. This is easy to demonstrate in the successor representation [16], a model closely related to ours. To behave similarly, the present model would require additional constraints to ensure the basis functions corresponding to different mazes are interchangeable, but this would be one route toward explaining shortcut phenomena in this framework. More generally, because the present proposal uses a state transition model, implicitly, to generate a basis function that is then used with model-free learning [see also 16], [83], [84], it resembles something of a cooperative hybrid of model-free and model-based techniques somewhat different from the competitive approaches suggested elsewhere [78].

## Materials and Methods

### Value functions and spatial reinforcement learning

We simulate value function learning in a gridworld spatial navigation task in order to compare linear function approximation over several different spatial basis sets [8]. Our model learns to estimate the value function over states (i.e., positions in the grid), defined in the standard way as the expected future discounted reward:
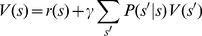
(1)


To simplify notation, we omit the dependence of these quantities on the action policy throughout. The model learns approximations to these values by learning a set of N linear weights *w_1…N_* for N spatial basis functions *φ_1…N_(s)* defined over the entire state space. The estimated value is thus:
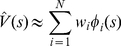
(2)


We use a simple temporal-difference algorithm with eligibility traces [8], [85] to learn weights. Specifically, at each run upon visiting state s receiving reward *r(s)* and transitioning into state *s′*, for each basis *φ_i_*, weights *w_i_* are updated at each time step using the following algorithm:
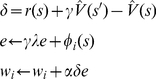
(3)


This is just the version of the familiar TD(λ) rule for linear value function approximation, with free parameters α (learning rate), *λ* (trace decay rate), and γ (discount factor).

### Gridworld simulations

We tested the model in 20-by-20 (M = 400 states) gridworlds in which the agent could move in any of the four cardinal directions, unless a wall blocked such a movement. Agents were started at a random location (i.e. state) at each trial, and had to reach the goal state, which was the only state with a reward, *r(s)* = 1. Individual trials ended when the agent reached the goal state, which was absorbing, or the maximum number of actions allowed, which was 500.

For simplicity, as described above the agent learns the value function over states and uses this to guide actions toward the goal, rather than directly learning the full Q-function over states and actions. This is because, in a spatial gridworld task, the state-action-state transition model is transparent, so we assume the agent evaluates the value 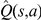 of each action in a state as the value 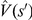 of the appropriate neighboring state [86]. Since the computation of Q involves a single step of what amounts to model-based lookahead, the approach is not as purely model-free as standard Q-learning or actor-critic algorithms. As with eligibility traces, we include this elaboration because it slightly improves generalization between states and actions, and might thus reduce the need for the sorts of basis-function-based generalization mechanisms we argue for.

The agent chooses actions according to a softmax policy, i.e. 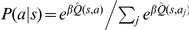, where actions unavailable (due to walls) are not considered and *β* is the inverse temperature that balances the amount of exploration and exploitation in action selection. For these simulations, the inverse temperature was fixed to *β* = 80 (a factor calibrated to provide a reasonable explore/exploit balance in choice probabilities given the scale of the action values learned). To maintain such balance, because each gridworld had a different distance between the goal state and other states, for each environment the discount factor was scaled to *γ* = 0.9*^d/c^* so that each gridworld had the same value range. Here, *d* is the shortest maximum distance from any state to the goal, across all gridworlds tested, and *c* is the maximum interstate distance for a given gridworld (range 26 to 105 states). In order to compare fairly the different basis functions, the learning rate *α* was chosen for each condition and each basis set to minimize the mean number of steps to termination over a fixed number of trials, using a grid search in the range [0,1]. All simulations and analyses were performed using Matlab (Natick, MA).

### Basis functions

We compare the model's learning using several different linear basis sets. Each basis is an M (states)×N (basis functions) matrix, with each column *φ_i_* defining a function over the states. Bases were constructed as below, and lastly each row of the matrix was normalized by its L_2_ norm. This ensures that the learning rate parameter *α* in the update rule (Equation 3) has a consistent interpretation (as a fractional stepsize) between different states and basis sets.

#### Tabular

The tabular basis is the M-by-M identity matrix, with one function corresponding uniquely to each state. It is easy to verify that using the identity basis that the value prediction and update equations (Equations 2 and 3, respectively) reduce to standard TD(λ) learning. In other words, the tabular basis is 1 at the current state and zero for all other states, thus the learned weights correspond directly to the values learned through standard TD(λ).

#### Place cell

We used isotropic 2D Gaussian basis functions at different standard deviations to model a multiscale place cell basis. Such a representation ignores the possibility that individual basis functions have multiple fields [e.g. 87], a condition we explore using a grid cell-like basis. Each Gaussian was evaluated over all x–y locations in the grid, where a given pair of coordinates corresponded to a single, unique state. Standard deviations were chosen to be 0.25, 0.15, 0.1, and 0.075 (expressed as fractions of the environment width, i.e. 20 states), such that the scales of the place cell firing fields roughly equaled the scales of individual nodes in the grid cell basis (see below). The center locations were evenly tiled in the gridworld's x–y coordinates, with the smaller functions distributed more densely (with 25, 49, 100, and 225 functions going from large to small scale) to produce a regular tiling of the state space. We also included a constant function, for a total of 400 bases.

#### Grid cell

We used the sum of three 2D spatial cosine waves to model a hexagonal grid cell-like basis, akin to previous models of grid cell responses [12], [13]. Following the approach of Blair et al. [12] a given basis function was represented as:
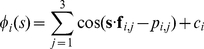
(4)


Here, the state ***s*** is expressed as a 2-vector of x–y coordinates on the gridworld; and a particular basis function *φ_i_* is defined by its phases p_i,j_, orientation *θ_i_*, and spacing λ_i_. Together, the grid orientation and spacing determine the vectors **f**_i,j_ onto which the planar cosine wave is projected. In particular, to produce a given grid orientation, *θ_i_*, the directions of the three vectors **f**_i,j_ are taken as *θ_i_*+π/2, *θ_i_*−π/6, and *θ_i_*+π/6. The vectors 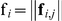 determine the periodicity of a given grid cell according to f*_i_* = 4π/(λ_i_3^0.5^) [12], [36], [39], where λ_i_ controls the space between simulated firing fields. For the waves to interfere constructively and produce a grid pattern, the three phases relate as *p_i,1_*+*p_i,2_* = *p_i,3_*.

We produced a basis set of 400 grid cell-like functions, using all combinations of four orientations θ_i_ (0, π/12, π/6, and π/4), four node spacings λ_i_ (4/(3*n*) environment widths for integers n = 1–4), and 25 different spatial phases evenly sampling the 2D space of phases p_i,1_ and p_i,2_ each between 0 and 2π. We also included a constant function, for a total of 401 bases. Finally, we ensured that the basis functions were non-negative (directly representable with firing rates) by adding an appropriate constant 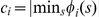. For all basis sets used (tabular, place cell, and grid cells), the weights for each basis function were learned independently.

#### Geodesic transformation

To modify basis sets to respect the wall layout of a particular grid task, the Euclidean x–y coordinates for each state were transformed such that their pairwise distances approximately reflected geodesic distances (i.e. distances along paths that respect boundaries) in the gridworld. The basis functions were then evaluated at these transformed coordinates. Specifically, coordinates were transformed in a manner analogous to the ISOMAP algorithm [54]. Floyd's Algorithm [88] was used to generate an M-by-M dissimilarity matrix, containing for each pair of states, the shortest-path distance (measured as the number of states) between them along the state adjacency graph. For the gridworlds shown in Figure 8, there are disconnected components on the state graph, which implies infinite geodesic distances between components and causes the next step of multidimensional scaling to be inestimable. To maintain the environment's integrity, we capped these infinite pairwise distances at their corresponding Euclidean distances.

Next, we estimated a set of Euclidean coordinates (i.e., an x–y pair for each of the M states) whose Euclidean inter-state distances approximated the geodesic distance matrix. This was accomplished by applying non-classical multidimensional scaling (Matlab, mdscale) to the dissimilarity matrix, using Sammon's nonlinear stress criterion [89] as the objective function. Insofar as these new coordinates differ from the original geodesic coordinates for a state, they reflect the distorted geodesic geometry. Using this transformed set of x–y coordinates, we then reevaluated the grid cell-like and place cell-like basis sets using the same sets of parameters (phase, spacing, and orientation) as in the Euclidean cases. Note that we specify field size as a fraction of environment width, and this remapping may stretch the environment. In this case, we scaled bases as fractions of the maximum of environment width or height, thus producing a basis scaled appropriately for the transformed environment.

We computed this transformation once for each environment, producing a static basis set over which to perform reinforcement learning. Realistically, the animal would have to learn the state transition function (i.e., the location of barriers) in order to compute the basis, and the firing fields would be expected to change as this state transition model was learned. However, since in our environments obstacles are sparse and observable from a distance, the true transition matrix (and the basis implied) should be entirely learned during the first trial in any of our environments.

#### Ground truth

Ground-truth value functions were computed for the optimal policy using dynamic programming over a tabular basis.
